# Quantifying remobilization of pre-existing nitrogen from cuttings to new growth of woody plants using ^15^N at natural abundance

**DOI:** 10.1186/1746-4811-9-27

**Published:** 2013-07-12

**Authors:** Lee A Kalcsits, Robert D Guy

**Affiliations:** 1 Department of Forest Sciences, University of British Columbia, 2424 Main Mall, Vancouver, BC V6T1Z4, Canada

**Keywords:** Nitrogen remobilization, Poplar, δ^15^N

## Abstract

**Background:**

For measurements of nitrogen isotope composition at natural abundance, carry-over of pre-existing nitrogen remobilized to new plant growth can cause deviation of measured isotope composition (δ^15^N) from the δ^15^Nof newly acquired nitrogen. To account for this problem, a two-step approach was proposed to quantify and correct for remobilized nitrogen from vegetative cuttings of *Populus balsamifera* L. grown with either nitrate (δ^15^N = 58.5‰) or ammonium (δ^15^N = −0.96‰). First, the fraction of carry-over nitrogen remaining in the cutting was estimated by isotope mass balance. Then measured δ^15^N values were adjusted for the fraction of pre-existing nitrogen remobilized to the plant.

**Results:**

Mean plant δ^15^N prior to correction was 49‰ and −5.8‰ under nitrate and ammonium, respectively. Plant δ^15^N was non-linearly correlated to biomass (r^2^ = 0.331 and 0.249 for nitrate and ammonium, respectively; P < 0.05) where the δ^15^N of plants with low biomass approached the δ^15^N of the pre-existing nitrogen. Approximately 50% of cutting nitrogen was not remobilized, irrespective of size. The proportion of carry-over nitrogen in new growth was not different between sources but ranged from less than 1% to 21% and was dependent on plant biomass and, to a lesser degree, the size of the cutting. The δ^15^N of newly acquired nitrogen averaged 52.7‰ and −6.4‰ for nitrate and ammonium-grown plants, respectively; both lower than their source values, as expected. Since there was a greater difference in δ^15^N between the carried-over pre-existing and newly assimilated nitrogen where nitrate was the source, the difference between measured δ^15^N and adjusted δ^15^N was also greater. There was no significant relationship between biomass and plant δ^15^N with either ammonium or nitrate after adjusting for carry-over nitrogen.

**Conclusion:**

Here, we provide evidence of remobilized pre-existing nitrogen influencing δ^15^N of new growth of *P. balsamifera* L. A simple, though approximate, correction is proposed that can account for the remobilized fraction in the plant. With careful sampling to quantify pre-existing nitrogen, this method can more accurately determine changes in nitrogen isotope discrimination in plants.

## Background

Measurements of nitrogen isotope composition at natural abundance (δ^15^N-the ^15^N:^14^N ratio of a sample relative to the isotope ratio of a known international standard (air N_2_)), expressed in *per mil*, may be affected by remobilization of previously acquired nitrogen into new plant growth. To determine the δ^15^N of newly acquired nitrogen, the carry-over of pre-existing nitrogen must be considered. The use of δ^15^N at natural abundance has increased in plant physiology and ecology where small but tractable changes δ^15^N of plants or plant tissues can indicate changes in soil nitrogen sources or changes in nitrogen-use physiology [1-6]. Quantifying temporal changes in plant nitrogen sources in the field or plant nitrogen isotope fractionation requires confidence in the measurement of the δ^15^N of newly acquired nitrogen. When not working at the natural abundance level, as in many ^15^N enrichment experiments, knowledge of the precise isotopic composition of pre-existing nitrogen may not be necessary because newly acquired nitrogen will have a markedly different isotopic signature [3,7].

Carried-over pre-existing nitrogen in a plant can be remobilized to growing tissue. For example, during establishment of sexually and vegetatively propagated plants, stored nitrogen is remobilized from the propagule (seed or cutting) to the newly growing tissue as a nitrogen supply until roots are capable of supporting the nitrogen demands of the plant [7]. In woody perennials, nitrogen from senescing leaves accumulates in stem tissue during autumn to be re-used by developing tissues during regrowth in the spring [8,9]. Nitrogen transported to new shoot and/or root growth come from vegetative storage proteins kept over the dormant period [10]. Vegetative storage proteins can account for a sizeable fraction of total nitrogen in dormant stem tissue [11,12]. These vegetative storage proteins, in addition to other mobile nitrogen fractions in stem tissue can be remobilized to shoots or roots of plants. If the δ^15^N of remobilized nitrogen is different from the δ^15^N of newly acquired nitrogen, it may influence the measured δ^15^N of new growth.

Although the δ^15^N of carried over nitrogen may provide an isotopic signature to compare against new growth δ^15^N, within a bulk nitrogen pool (e.g., a cutting), there may be differences in δ^15^N between various amino acids and other nitrogen-containing compounds [13-15]. This may impact the δ^15^N of remobilized nitrogen relative to the δ^15^N of the bulk cutting prior to new growth if the δ^15^N of the remobilized fraction is different from the non-remobilized fraction of the cutting. However, if much of the carried-over pre-existing nitrogen contributes to the growth of new plant tissue, the δ^15^N of the remobilized nitrogen should resemble the δ^15^N of the bulk source tissue because many, and certainly the most predominant, nitrogen-containing compounds (e.g. vegetative storage proteins) will contribute to remobilized nitrogen from the source tissue (cutting) to new growth. If there was any isotope discrimination associated with remobilization itself, as consumption of a nitrogen pool approaches completion, the transferred fraction (the product) would then approach the δ^15^N of the stored fraction (the source).

Here, a two-step mass balance approach is proposed to account for carry-over nitrogen remobilized from pre-existing nitrogen pools to growing tissues in plants using *Populus balsamifera* L. (balsam poplar) grown with either ammonium or nitrate. The two nitrogen sources bracketed the δ^15^N of pre-existing N, with the nitrate being enriched in ^15^ N relative to the cutting and the ammonium being depleted relative to the cutting. Therefore, if carried-over nitrogen from pre-existing nitrogen has an appreciable effect on newly assimilated nitrogen isotope values, opposing relationships with biomass should be evident. Poplars in general have long been used as model systems in tree biology and genetics, and *Populus trichocarpa* Torr. & Gray, (syn: *P. balsamifera* ssp. *trichocarpa*) was the first woody species to have its genome completely sequenced [16]. The approach described here is applicable not just to plants grown from cuttings, but also, with little adjustment, to plants grown from seed.

## Results

### δ^15^N, growth and nitrogen concentration

**Figure 1:**
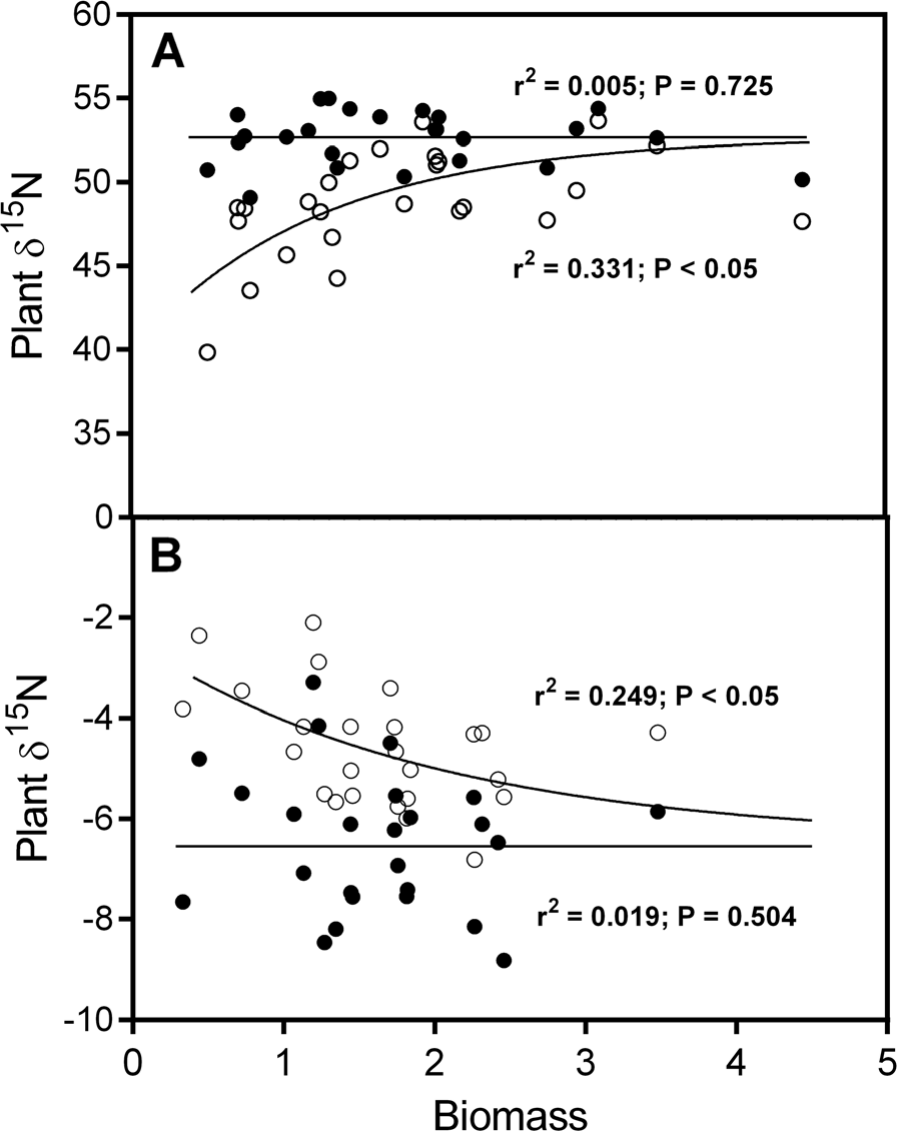
**Unadjusted (open symbols) and adjusted (closed symbols) δ**^**15**^**N for new growth of the whole plant plotted against biomass for *Populus balsamifera *L. grown with either nitrate (A) or ammonium (B).** Each data point represents a genotypic mean (N = 3). Correlation coefficients and P-values are placed near each line.

Both ammonium and nitrate-grown plants were depleted in ^15^ N relative to the source (Table 1). Under nitrate, mean root, stem and leaf δ^15^N was 44.4, 47.1 and 50.1‰ whereas for ammonium, these values were −8.6, -6.6 and −5.1‰, respectively. The vegetative cuttings used to propagate the plants were not different between sources and had a mean δ^15^N of 0.92‰ at the start of the experiment. During plant growth, the δ^15^N of the cuttings moved towards and/or past the isotope signature of the source (Table 1). For nitrate-grown plants, cuttings became enriched (23.5‰) and for ammonium-grown plants, cuttings became depleted (−3.1‰) relative to their starting isotopic composition (P < 0.05). Genotypic means for whole plant biomass ranged from 0.51 to 4.46 g and 0.33 to 3.48 g under nitrate and ammonium, respectively (Table 2). There was a significant correlation between genotype means for biomass on nitrate versus ammonium (r = 0.54, P < 0.05) whereby clones that grew well on one source also grew well on the other. The range in mean genotype biomass provided a range of isotopic dilutions to determine the influence of carried-over pre-existing nitrogen on whole-plant and organ level δ^15^N. When the unadjusted δ^15^N for new growth of the whole plant was plotted against biomass, there was a significant non-linear relationship (r^2^ = 0.331 and 0.249 for nitrate and ammonium, respectively; P < 0.05) (Figure 1). The unadjusted δ^15^N for new growth of the whole plant for genotypes with low biomass were closer to the δ^15^N of cutting nitrogen. In contrast, the δ^15^N of genotypes with high biomass were closer to the δ^15^N of newly acquired nitrogen, which resembles the source but does not equal it due to isotope discrimination that occurs during uptake and assimilation. Nitrogen concentration was not different between cuttings before the start of the experiment, but was significantly greater at the end of the experiment when grown with ammonium as compared to nitrate (Table 3). Root nitrogen concentration was not significantly different between sources but stem and leaf nitrogen concentrations were greater when grown with ammonium (P < 0.05).

**Table 1:** **Mean measured and, for new tissues, adjusted root, stem, leaf and cutting δ**^**15**^**N values (‰ ± SE; N = 75) of *Populus balsamifera *L. hydroponically grown with nitrate (58.5‰) or ammonium (−0.96‰)**

	**Nitrate**	**Ammonium**
	**Measured δ**^**15**^**N**	
Plant	49.04 ± 0.43	−5.81 ± 0.16
Root	44.42 ± 0.42	−8.59 ± 0.15
Stem	47.08 ± 0.40	−6.62 ± 0.18
Leaf	50.10 ± 0.44	−5.07 ± 0.15
Cutting_*start*_	0.87 ± 0.35	0.97 ± 0.31
Cutting_*end*_	23.47 ± 0.88	−3.10 ± 0.23
	**Adjusted δ**^**15**^**N**	
Plant	52.68 ± 0.26	−6.43 ± 0.19
Root	47.87 ± 0.28	−9.45 ± 0.19
Stem	50.76 ± 0.26	−7.29 ± 0.19
Leaf	54.01 ± 0.25	−5.59 ± 0.18

**Table 2:** **Mean root, stem, leaf, cutting and whole plant biomass (g ± SE; N = 75) of *Populus balsamifera *L. hydroponically grown with nitrate or ammonium**

	**Biomass (g)**	
	**Nitrate**	**Ammonium**
Root	0.18 ± 0.02	0.20 ± 0.01
Stem	0.40 ± 0.03	0.29 ± 0.02
Leaf	1.28 ± 0.09	1.15 ± 0.07
Cutting_start_	0.49 ± 0.03	0.54 ± 0.04
Cutting_end_	0.67 ± 0.06	0.65 ± 0.05
Whole Plant	1.86 ± 0.14	1.64 ± 0.10

**Table 3:** **Mean nitrogen concentrations (μmol g dw**^**-1**^ **± SE; N = 75) of roots, stems, leaves and cuttings of *Populus balsamifera *L. hydroponically grown with nitrate or ammonium**

	**Tissue nitrogen concentration (μmol g dw**^**-1**^**)**	
	**Nitrate**	**Ammonium**
Root	2.50 ± 0.04	2.55 ± 0.03
Stem	0.41 ± 0.01	0.76 ± 0.03
Leaf	1.84 ± 0.03	2.19 ± 0.04
Cutting_*start*_	0.83 ± 0.01	0.83 ± 0.02
Cutting_*end*_	0.59 ± 0.03	0.71 ± 0.02

### Carry-over nitrogen in new plant growth is dependent on the size of the nitrogen pool in the cutting relative to the nitrogen pool in the new growth

**Figure 2:**
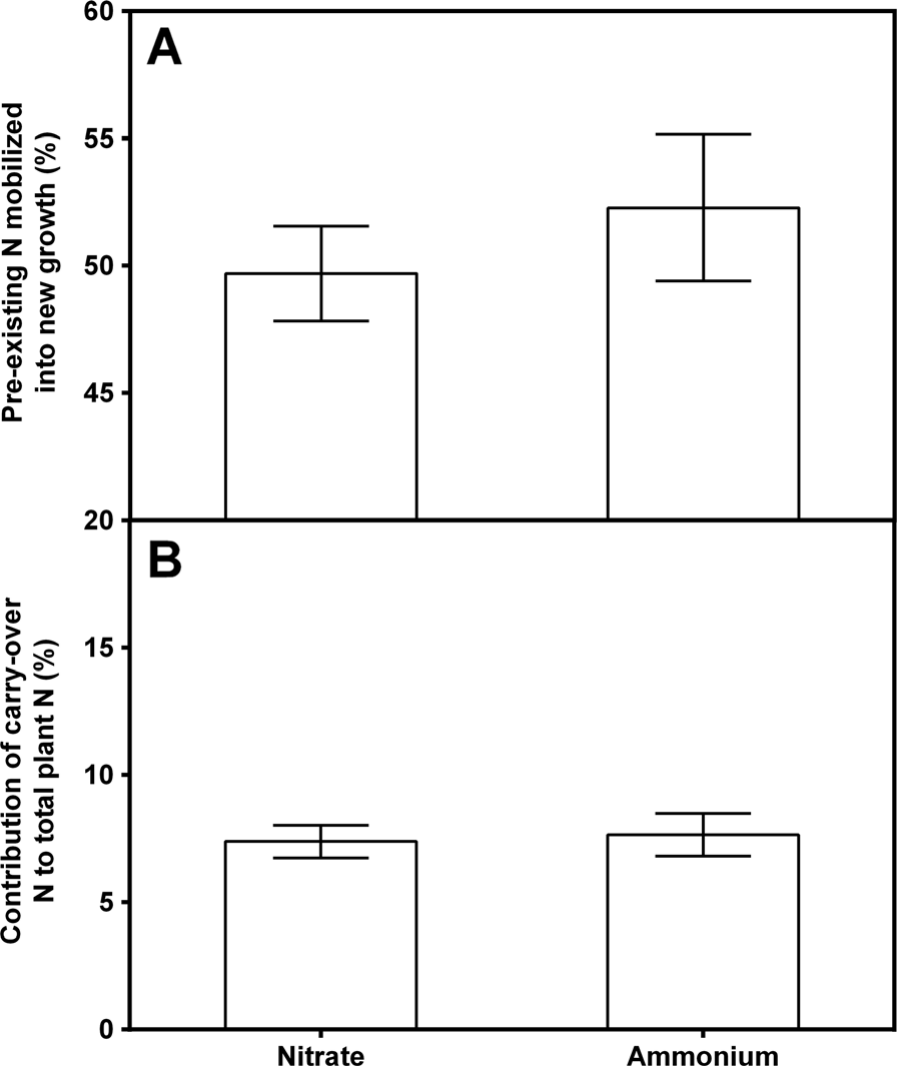
**Per cent of pre-existing nitrogen remobilized into new growth (A) and per cent contribution of carried-over pre-existing cutting nitrogen to total plant nitrogen (B) in *Populus balsamifera *L. grown with either nitrate or ammonium.** Bars are means ± SE (N = 75).

**Figure 3:**
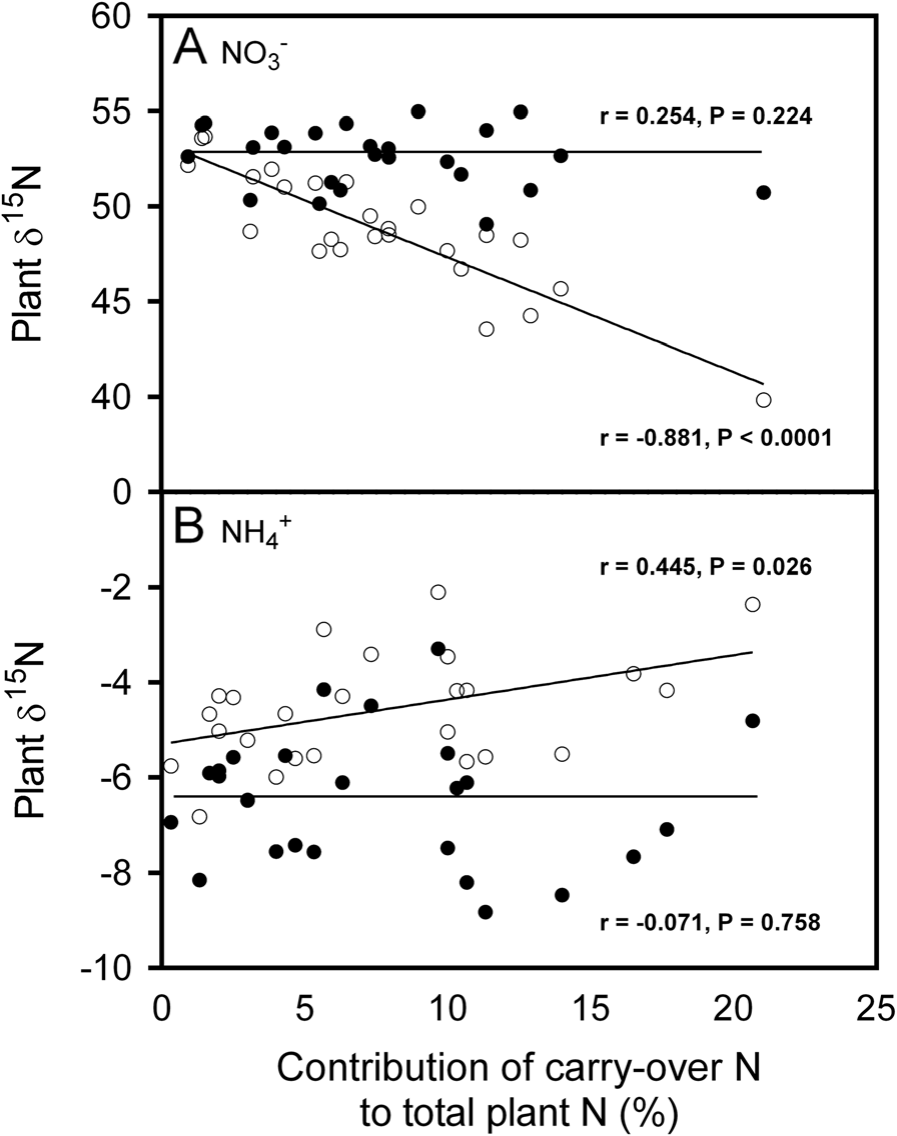
**Unadjusted (open symbols) and adjusted (closed symbols) δ**^**15**^**N for new growth of the whole plant plotted against the per cent contribution of carry-over nitrogen to new growth of *Populus balsamifera *L. supplied with either nitrate (A) or ammonium (B).** Each data point represents a genotypic mean (N = 3). Correlation coefficients and P-values are placed near each line.

The δ^15^N of vegetative cuttings at the end of the experiment was approximately halfway between the starting cutting δ^15^N and the δ^15^N of newly formed organs, indicating that some considerable portion of the original nitrogen was retained within the cuttings. From equation 4, the non-remobilized fraction of the nitrogen contained within the cuttings at the end of the experiment averaged 50% and 52% for nitrate and ammonium, respectively and was not significantly different. Therefore, approximately half of the starting nitrogen was remobilized for new growth (Figure 2). The mean quantity of nitrogen remobilized was 3.2 mg but was dependent on the size of the cutting (r = 0.057; P < 0.05; data not shown). There was no source effect on the proportion of carry-over, which averaged 8% but decreased non-linearly from 21% to 1% with the biomass of new growth (not shown). The difference between source δ^15^N and the cutting δ^15^N at harvest was greater for nitrate than ammonium (Table 1), causing carry-over nitrogen to have a greater influence on measured plant δ^15^N under nitrate (Figure 3A) than ammonium (Figure 3B).

### Relationships between plant δ^15^Nand biomass were not significant after accounting for carry-over of pre-existing nitrogen

There were significant linear relationships between the contribution of nitrogen carried-over from the cutting and the unadjustedδ^15^N for new growth of the whole plant (P < 0.05) (Figure 3). Relative to the measured δ^15^N of each corresponding organ, newly assimilated nitrogen was more depleted in ^15^ N under ammonium and more ^15^ N enriched under nitrate. After accounting for the presence of remobilized nitrogen, the adjusted root, stem and leaf δ^15^N values were 47.87, 50.76 and 54.01‰ and −9.45, -7.29 and −5.59‰ for nitrate and ammonium, respectively. This represented an approximate shift of 3.5 to 4‰ and 0.5 to 0.8‰ for nitrate and ammonium grown plants, respectively. There was no residual relationship between biomass and newly acquired plant δ^15^N (i.e., non-significant regressions in Figure 3) indicating that the carry-over nitrogen was well accounted for.

### The δ^15^N of remobilized nitrogen to new growth is not significantly different than the δ^15^Nof the cutting

When cuttings of randomly selected *Populus trichocarpa* Torr. & Gray genotypes were flushed without exogenous sources of nitrogen, the nitrogen isotope composition of remobilized nitrogen was not significantly different than the isotope composition of complementary cuttings sampled prior to bud flush (Table 4). The isotope composition of the bud was significantly greater (P < 0.05) than the cutting stem (2.44 vs 2.11‰) and accounted for approximately 10% of the nitrogen in the vegetative cuttings. After flushing, the isotope composition of the shoot was not significantly different than the cutting stem (P > 0.05) and similar to our estimates using isotope mass balance, approximately 50% of the nitrogen from the cutting stem and bud was allocated to the shoot (data not shown). Since a greater portion of nitrogen was allocated to the shoot after flushing, there was a corresponding 40% decrease in the nitrogen content of the cutting stem after flushing. Although there was significant genotypic variation in the isotope composition of the cuttings, there was no significant difference in the amount of nitrogen remobilized from the cuttings (P > 0.05).

**Table 4:** **Nitrogen isotope composition of vegetative cuttings, pre-flush and post-flush (‰ ± SE; N = 5 except for roots (N = 2-5) where replicates were combined when root biomass was too low for analysis), for four randomly selected *Populus trichocarpa *Torr.** &**Gray genotypes flushed in ddH**_**2**_**O containing no exogenous sources of nitrogen for 21 days**

	**Pre-flush cutting**		**Post-flush cutting**		
**Genotype**	**CuttingStem δ**^**15**^**N**	**Bud δ**^**15**^**N**	**CuttingStem δ**^**15**^**N**	**Shoot δ**^**15**^**N**	**Root δ**^**15**^**N**
LILD 26-3	2.34 ± 0.17	1.97 ± 0.27	2.11 ± 0.34	2.53 ± 0.13	3.08 ± 0.02
LILD 26-5	1.68 ± 0.16	1.97 ± 0.24	1.63 ± 0.36	1.63 ± 0.06	2.46 ± 0.04
TOBA 23-3	3.35 ± 0.26	2.25 ± 0.18	2.82 ± 0.46	2.88 ± 0.08	3.02 ± 0.06
PHLA 22-1	2.35 ± 0.17	2.28 ± 0.16	2.34 ± 0.37	2.41 ± 0.08	2.20 ± 0.26

## Discussion

The objective was to quantify and correct for carry-over nitrogen from cuttings in new growth of *P. balsamifera* hydroponically grown under steady-state conditions. Here, it was shown that nitrogen remobilized from cuttings influences plant δ^15^N. Carry-over nitrogen would be inconsequential for plants grown from relatively small seeds or in longer-term experiments, where the total accumulated nitrogen pool represents almost all of the nitrogen within the plant. However, in cases where biomass accumulation is low relative to the size of the propagule, without correction, deviation of measured δ^15^N from the δ^15^N of newly acquired nitrogen can lead to misinterpretations in physiological and ecological studies. It has been suggested that there are carry-over effects contributing to interannual variability in carbon isotope signatures (δ^13^C) in woody plants [17,18] but as far as we know, aside from [19], this is the first attempt to quantify the carry-over effect on nitrogen isotope composition of new growth in woody plants.

Accounting for remobilized nitrogen from the cutting to the root, stems and leaves required a two-step approach. Since vegetative cuttings are a part of the stem, nitrogen contained within the cutting should be combined with the general stem nitrogen pool. However, there is a certain fraction of nitrogen in the vegetative cutting that was not mobilized and therefore, needs to be estimated to calculate the corresponding amount of cutting nitrogen that was remobilized into the new growth. In the present study, and noting that some isotope discrimination is expected during nitrogen uptake and assimilation [4,6,14,20], the presence of non-remobilized nitrogen in the cutting was indicated by a difference between stem and cutting δ^15^N that was weighted towards the source δ^15^N (Table 1). Previous work has indicated that there is often a fraction that is not remobilized to sink tissue [8]. Once non-remobilized nitrogen and the proportion of cutting nitrogen that is part of the stem nitrogen pool had been partitioned, the amount of nitrogen mobilized into the plant nitrogen pool was quantified. To make this partitioning calculation, it was assumed that new nitrogen in the cutting was mixed with remobilized (carried-over) nitrogen from the cutting similar to that of stem tissue. Since there was influx of newly assimilated nitrogen into the vegetative cutting, as indicated by a growth dependent change of cutting δ^15^N towards the source δ^15^N, it would be reasonable to assume there is a certain degree of mixing of nitrogen within the mobile stem nitrogen pool.

Variation in the difference in δ^15^N between measured and newly acquired nitrogen may, in part, be a consequence of assuming that the distribution of carry-over nitrogen was proportionate to the nitrogen allocation between roots, stems and leaves. Dong S, 2004 [7] reported that approximately 30% of remobilized nitrogen was allocated to the root and 70% was allocated to the shoot in vegetatively propagated poplar, which favors shoots over roots slightly less than our results. Although total nitrogen contents and biomass of roots and shoots were not reported in [7], if the root:shoot ratios were similar to our results, then this would be consistent with a proportional allocation of remobilized nitrogen to those organs. By assuming proportionate allocation of carry-over nitrogen, we acknowledge that there may be some error associated with this assumption but the overall effect of the associated error is small. In situations where carry-over nitrogen accounted for a large portion of plant nitrogen, unequal allocation could have an appreciable effect. However, in our case where, in most cases, carry-over nitrogen accounted for less than 15% of plant nitrogen, the influence on adjusted isotopic composition for roots, stems and leaves would be mostly inconsequential.

Another assumption made was to equate the δ^15^N of remobilized nitrogen with the δ^15^N of the cutting at the start of experiment. To validate this assumption, vegetative cuttings from four randomly chosen genotypes from a common garden experiment for *P*. *trichocarpa* were grown without exogenous nitrogen for 21 days. The estimates of the amount of nitrogen remobilized from the cutting to the new growth were similar to those obtained using our proposed isotopic mixing model and correction. Using a different approach, [19] reported that there was no difference in remobilized δ^15^N and stored δ^15^N, and that no fractionation occurred during remobilization. Although biochemical variation in δ^15^N occurs in plants [13-15] and these variations may contribute to error to our approach, our results indicate that it would seem to be small relative to the correction applied.

The proportion of soluble nitrogen in cuttings may limit nitrogen remobilization which may provide an explanation for why approximately half of pre-existing nitrogen was not remobilized. During the dormant period, nitrogen storage proteins accumulate in stem tissue and the proportion of soluble, stored nitrogen increases [21-24]. Estimates of soluble nitrogen in stems of *Fraxinus excelsior* were between 60 and 70% during the dormant period [20]. Our natural abundance nitrogen isotope mass balance approach indicates that approximately 50% of total cutting nitrogen was remobilized to sink tissues (leaves, roots and stems). If the proportion of soluble nitrogen in *P. balsamifera* is similar, then only a small fraction of soluble nitrogen was not remobilized to new growth. For hybrid poplar, between 60 and 70% of nitrogen in cuttings was remobilized into actively growing tissue [7]. This proportion was comparable with estimates of remobilization of nitrogen from source tissue (leaves) of *Brassica napus* to sinks (developing siliques) [25].

The use of two separate nitrogen sources with δ^15^N values both greater and lower than the δ^15^N of the cutting at the start of the experiment provided some validation for the correction. Previously, enriched nitrogen applied as ^15^ N allowed for the quantification of remobilized nitrogen into growing plant tissue [7]. In the present study, newly acquired nitrogen was approximately 5 to 10‰ depleted relative to the cutting when plants were grown on ammonium (δ^15^N = −0.96 ‰), and 50 to 55‰ enriched when plants were grown on nitrate (δ^15^N = 58.5 ‰). This contrast in the isotopic composition of two sources of newly acquired nitrogen, relative to nitrogen that previously existed in the cutting, produced negative and positive non-linear relationships between biomass and the unadjusted plant δ^15^N. Here, after correction, biomass had no impact on adjusted plant δ^15^N for either source and any observed relationship was primarily a result of nitrogen carried-over from the cutting. Our approach worked well either way, which should not be the case if the δ^15^N of remobilized nitrogen were consistently offset in one direction from the δ^15^N of bulk nitrogen.

## Conclusion

Here, we provided evidence that remobilized nitrogen carried-over from stem cuttings can affect measured δ^15^N of new growth in poplar, a perennial model plant. Carry-over nitrogen from pre-existing nitrogen pools needs to be considered when measuring δ^15^N at natural abundance. By applying a two-step mass balance correction, the contributions of carried-over pre-existing nitrogen to the nitrogen content and δ^15^N of new growth can be quantified and accounted for. This, in turn, will allow for a better assessment of isotope discrimination associated with the acquisition of new nitrogen from the rooting environment and could be helpful in physiology and ecology studies measuring nitrogen isotope composition at natural abundance to address questions relating to nitrogen use dynamics at the soil or plant level. The methods presented here are not only applicable to this particular species or set of conditions but, with careful sampling protocols, can be applied to other experimental systems that require the measurement of δ^15^N at natural abundance during plant growth and development.

## Methods

### Plant material and experimental design

First year branches of 25 genotypes of *Populus balsamifera* L. ranging from 51°N to 56°N from the Agriculture Canada Balsam Poplar (AgCanBaP) collection [26] were obtained from the AAFC-AESB Agroforestry Development Centre at Indian Head, Saskatchewan, Canada and stored at 4°C for approximately three months to fulfill chilling requirements. The five provenances reflected a climatic gradient for the species that extends from a prairie ecosystem to the boreal forest of the Canadian Shield; namely Outlook (OUT; 51.1°N, 106.2°W), Saskatoon (SKN; 2.2°N, 106.4 °W), Turtleford (TUR; 53.2°N, 108.3 °W), Cold Lake (CLK; 54.2°N, 110.1°W) and Gillam (GIL; 56.4°N, 94.7 °W). Two-node cuttings, approximately 6–8 cm long were weighed for fresh weight and arranged in a randomized complete block design with three blocks of two nitrogen treatments supplied as either 250 μM Ca(NO_3_)_2_ or 250 μM (NH_4_)_2_SO_4_. Plants were grown for 45 days in a hydroponics solution until harvest. Complementary samples of each genotype were collected (N = 3) to determine initial isotope composition and nitrogen concentration.

### Hydroponics system

The hydroponics system was comprised of six 1000 L containers lined with rubber pond liner material (Firestone, Nashville, TN, USA) set up in a greenhouse under ambient light conditions supplemented by sodium halide lighting (600 μmol m^-2^ s^-1^ PPFD) and 18/6 photoperiod. Temperatures in the greenhouse were maintained at between 20 and 24°C. Each container was fitted with a floating Perspex “raft” that held up to 32 plants. Unused plugs in the raft and the rest of the container were covered with black polythene to prevent algal growth. The hydroponics solution was a modified 1/10^th^ strength Johnson’s solution [27] supplemented with either 250 μM Ca(NO_3_)_2_ or (NH_4_)_2_(SO_4_). Final nutrient composition, excluding nitrogen, was: 200 μM KH_2_PO_4_, 200 μM K_2_SO_4_, 100 μM MgSO_4_, 100 μM CaSO_4_, and micronutrients: 5 μM Cl, 2.5 μM B, 0.2 μM Mn, 0.2 μM Zn, 0.1 μM Mo, 0.05 μM Cu, and 50 μM Fe^2+^. Containers were fitted with 20 L per minute centrifugal pumps, to circulate and aerate media. Solutions were monitored periodically for oxygen levels, pH and temperature. Powdered calcium carbonate (CaCO_3_) was added to buffer pH in the range of 6–7.5. Media NH_4_^+^ and NO_3_^-^ concentrations were assayed using the phenol: hypochlorite [28] and perchloric acid [29] methods, respectively. The solution was completely replaced every 14 days to ensure that there was no substantial decrease (>10%) in concentration of nitrate or ammonium over time that could increase the solution δ^15^N.

### Sampling and natural abundance isotope analysis

After 45 days of growth, plants were separated into leaves, stems, roots and the original cutting. Samples were flash frozen in liquid nitrogen and stored at −80°C until samples could be freeze-dried at −50°C for two days. Once dried, roots, leaves, stems, cuttings and the cuttings collected at the start of the experiment were weighed. Samples were then ground to a fine powder using a mortar and pestle and then a ball mill (Fritsch Laborgeratebau, Terochem Scientific). Subsamples of 3 ± 0.1 mg were weighed into tin capsules (Elemental Microanalysis Ltd., 8×5 mm, D1008) and analyzed for nitrogen concentration and δ^15^N on a PDZ Europa ANCA-GSL elemental analyzer interfaced to a PDZ Europa 20–20 isotope ratio mass spectrometer (Sercon Ltd., Cheshire, UK) (University of California Stable Isotope Facility, Davis, CA). Isotopic composition is expressed as δ^15^N:

(1) $$\delta^{15}N=\left(\frac{R_{\mathrm{sample}}}{R_{\mathrm{standard}}}-1\right)*1000$$

where, R_*sample*_ is the ^15^ N/^14^ N isotope ratio of the sample and R_*standard*_ is the isotope ratio of a known standard (air). The δ^15^N values of the ammonium and nitrate salts used for the growth media were −0.96 and +58.5‰, respectively.

### Correcting for carried-over pre-existing cutting nitrogen in growing plant organs

The δ^15^N and nitrogen concentration of the cutting after harvest was compared to the δ^15^N and nitrogen concentration of the cutting before the start of the experiment to correct for pre-existing nitrogen remobilized to the actively growing plant. Nitrogen content of the cutting at harvest *(*N_*end*_*)* can be calculated as:

(2) $$N_{\mathrm{end}}=\mathrm{Biomas}s_{\mathrm{cutting}\;\mathrm{end}}\times {\left[N\right]}_{\mathrm{cutting}\;\mathrm{end}}$$

where, Biomass_*cutting end*_and [N]_*cutting end*_ are the biomass and nitrogen concentration of the cutting at harvest. Since the cutting was part of the growing stem, dry mass accumulated in the cutting. The initial dry mass of the cutting (Biomass_*cutting start*_) was estimated from the fresh mass of the cutting at the start of the experiment and the mean dry mass content of the cuttings sampled at the start of the experiment (0.584). From this, nitrogen content at the start of the experiment (N_*start*_) was estimated as:

(3) $$N_{\mathrm{start}}=\mathrm{Biomas}s_{\mathrm{cutting}\;\mathrm{start}}\times {\left[N\right]}_{\mathrm{start}}$$

where, [N]_*start*_ is the mean nitrogen concentration of the cuttings at the start of the experiment. Assuming that the isotopic composition of a cutting at harvest (δ^15^N_*end*_) is a mixture of stem δ^15^N (δ^15^N_*stem*_) (containing a portion of remobilized nitrogen) and non-remobilized nitrogen remaining in the cutting (δ^15^N_*start*_), the fraction of non-remobilized (*f*_*nonremobilized*_) nitrogen can be estimated as:

(4) $$f_{\mathrm{nonremobilized}}=\frac{\left(\delta^{15}N_{\mathrm{end}}-\delta^{15}N_{\mathrm{stem}}\right)}{\left(\delta^{15}N_{\mathrm{start}}-\delta^{15}N_{\mathrm{stem}}\right)}$$

The amount of nitrogen remobilized (N_*remobilized*_) to new growth can then be calculated as:

(5) $$N_{\mathrm{remobilized}}=N_{\mathrm{start}}-\left(N_{\mathrm{end}}\times f_{\mathrm{nonremobilized}}\right)$$

The proportion of carried-over pre-existing nitrogen in the general plant nitrogen pool that was carried over into new growth (*C*) is given by:

(6) $$C=\frac{N_{\mathrm{remobilized}}}{N_{\mathrm{plant}}}$$

where, N_*plant*_ is equal to the sum of all nitrogen contents in the roots, leaves and stems (including new and remobilized nitrogen allocated to the expanded cutting):

(7) $$\begin{array}{l} N_{\mathrm{plant}}=N_{\mathrm{leaves}}+N_{\mathrm{roots}}+N_{\mathrm{stem}}+N_{\mathrm{end}}\\ \;\times \left(1-f_{\mathrm{nonremobilized}}\right) \end{array}$$

Assuming that there is a proportionate distribution of remobilized nitrogen throughout the plant (i.e., *C* is the same for all plant organs), the mass balance equation showing the contributions of new and carry-over nitrogen to measured δ^15^N for roots, stems and leaves is:

(8) $$\begin{array}{l} \delta^{15}N_{\mathrm{unadjusted}}=\delta^{15}N_{\mathrm{carry}-\mathrm{over}}\times C+\delta^{15}N_{\mathrm{new}}\\ \;\times \left(1-C\right) \end{array}$$

where, δ^15^N_*unadjusted*_, δ^15^N_*carry-over*_ and δ^15^N_*new*_ are the measured isotopic compositions of each plant organ, the isotopic composition of remobilized nitrogen (equal to δ^15^N_*start*_), and the isotopic composition of newly assimilated nitrogen, respectively. Equation 8 can be rearranged to yield:

(9) $$\delta^{15}N_{\mathrm{new}}=\frac{\delta^{15}N_{\mathrm{measured}}-\delta^{15}N_{\mathrm{carry}-\mathrm{over}}\times C}{\left(1-C\right)}$$

This equation was used to obtain root, stem and leafδ^15^N values adjusted for the contribution of carry-over nitrogen from the cuttings.

### Statistical analysis

Two-way ANOVA was used to test for the fixed effects of nitrogen source and genotype on (1) biomass, (2) measured δ^15^N values, (3) the proportion of cutting nitrogen remobilized to the growing plant, (4) the proportion of nitrogen in new growth that is carry-over nitrogen, and (5) the adjusted δ^15^N values of plant, root and leaf tissues. The statistical model was as follows:

(10) $$Y_{\mathrm{ij}}=\mu +\alpha_{i}+\tau_{j}+\beta_{\mathrm{ij}}$$

where, *μ* is the overall mean response, *α*_*i*_ is the effect due to the genotype, *τ*_*j*_ is the effect due to the nitrogen source and *β*_*ij*_ is the effect due to any interaction between the genotype and nitrogen source. Analysis of variance procedure was carried out using Graphpad Prism 6 (La Jolla, CA, USA) to obtain estimates of the means followed by Tukey’s multiple comparison tests to separate means. Differences between treatments described as significant are those where P <0.05. Whole plant δ^15^N was plotted against biomass and per cent carry-over nitrogen to examine the influence on measured δ^15^N of genotypes grown using nitrate or ammonium. Non-linear regression was performed for the measured plant δ^15^N versus biomass using Graphpad Prism 6 to fit an exponential model to the data. Linear regression was performed on adjusted plant δ^15^N versus biomass and on both measured and adjusted plant δ^15^N versus the proportion of carry-over nitrogen in new plant growth.

### Flushing vegetative cuttings without addition of exogenous nitrogen

Vegetative cuttings from four randomly selected *P. trichocarpa* genotypes were selected from a common garden experiment and stored at 2°C until flushing. Complementary cuttings (N = 5) were selected where one was allocated for flushing and the cutting immediately adjacent was separated into bud and cutting stem and oven-dried at 60°C for four days. Cuttings allocated for flushing were randomly placed in a floating foam raft in ddH_2_O for 21 days. At harvest, plants were separated into cutting stem, shoot and roots and oven dried for 4 days at 60°C. Samples were then weighed, ground and analyzed for nitrogen content and isotope composition as described above. Samples were then analyzed using an Isoprime (GV Instruments) Isotope Ratio Mass Spectrometer (IRMS) coupled with an Elementar Vario EL Cube Elemental Analyzer (EA) (UBC Faculty of Forestry Stable Isotope Facility). t-tests were used to compare nitrogen content and isotope means between genotypes and plant parts (P = 0.05).

## Competing interests

The authors declare that they have no competing interests.

## Authors’ contributions

LAK conceived and carried out the nitrogen isotope discrimination experiments, developed the correction and drafted the manuscript. RDG participated in its design and helped to draft the manuscript. All authors read and approved the final manuscript.

## References

[B1] Comstock JP Steady-state isotopic fractionation in branched pathways using plant uptake of NO_3_^-^ as an example Planta 2001 214 220 234 10.1007/s004250100602 11800386

[B2] Evans RD Physiological mechanisms influencing nitrogen isotope composition Trends Plant Sci 2001 6 121 126 10.1016/S1360-1385(01)01889-1 11239611

[B3] Dawson TE Mambelli S Plamboeck AH Templer PH Tu KP Stable isotopes in plant ecology Ann Rev Ecol Sys 2002 33 507 559 10.1146/annurev.ecolsys.33.020602.095451

[B4] Pritchard ES Guy RD Nitrogen isotope discrimination in white spruce fed with low concentrations of ammonium and nitrate Trees-Struct Funct 2005 19 89 98 10.1007/s00468-004-0367-2

[B5] Houlton BZ Sigman DM Schuur EAG Hedin LO A climate driven switch in plant nitrogen acquisition within tropical forest communities P Natl Acad Sci USA 2007 104 8902 8906 10.1073/pnas.0609935104 PMC1885600 17502607

[B6] Kalcsits LA Guy RD Whole plant and organ level nitrogen isotope discrimination indicates modification of partitioning of assimilation, fluxes and allocation of nitrogen in knockout lines of *Arabidopsis thaliana* Physiol Plantarum 2013 In press 10.1111/ppl.12038 23414092

[B7] Dong S Cheng L Scagel CF Fuchigami LH Nitrogen mobilization, nitrogen uptake and growth of cuttings obtained from poplar stock plants grown in different N regimes and sprayed with urea in autumn Tree Physiol 2004 24 355 359 10.1093/treephys/24.3.355 14704145

[B8] Millard P Thomson CM The effect of the autumn senescence of leaves on the internal cycling of nitrogen for the spring growth of apple trees J Ex Bot 1989 40 1285 1289 10.1093/jxb/40.11.1285

[B9] Malaguti D Millard P Wendler R Hepburn A Tagliavini M Translocation of amino acids in the xylem of apple (*Malus domestica* Borkh.) trees in spring as a consequence of both N remobilization and root uptake J Exp Bot 2001 52 1665 1671 10.1093/jexbot/52.361.1665 11479331

[B10] Cooke JE Weih M Nitrogen storage and seasonal nitrogen cycling in *Populus*: bridging molecular physiology and ecophysiology New Phytol 2005 167 19 30 10.1111/j.1469-8137.2005.01451.x 15948826

[B11] Greenwood JS Stinissen HM Peumans WJ Chrispeels MJ *Sambucus nigra* agglutinin is located in protein bodies in the phloem parenchyma of the bark Planta 1986 167 275 278 10.1007/BF00391426 24241862

[B12] Gomez L Faurobert M Contribution of vegetative storage proteins to seasonal nitrogen variations in the young shoots of peach trees (*Prunus persica* L. Batsch) J Ex Bot 2002 53 2431 2439 10.1093/jxb/erf098 12432035

[B13] Werner RA Schmidt HL The *in vivo* nitrogen isotope discrimination among organic plant compounds Phytochemistry 2002 61 465 484 10.1016/S0031-9422(02)00204-2 12409013

[B14] Tcherkez G Natural ^15^N/^14^N isotope composition in C_3_ leaves: are enzymatic isotope effects informative for predicting the ^15^N-abundance in key metabolites? Funct Plant Biol 2011 38 1 12 10.1071/FP10091 32480857

[B15] Gauthier PPG Lamothe M Mahé A Molero G Nogués S Hodges M Tcherkez G Metabolic origin of δ^15^N values in nitrogeneous compounds from *Brassica napus* L. leaves Plant Cell Environ 2012 In Press 10.1111/j.1365-3040.2012.02561.x 22709428

[B16] Tuskan GA DiFazio S Jansson S Bohlmann J Grigoriev I Hellsten U Putnam N Ralph S Rombauts S Salamov A The genome of black cottonwood, *Populus trichocarpa* (Torr. & Gray) Science 2006 313 1596 1604 10.1126/science.1128691 16973872

[B17] Vaganov EA Schulze ED Skomarkova MV Knohl A Brand WA Roscher C Intra-annual variability of anatomical structure and δ^13^C values within tree rings of spruce and pine in alpine, temperate and boreal Europe Oecologia 2009 161 729 745 10.1007/s00442-009-1421-y 19653008 PMC2744769

[B18] Offermann C Ferrio JP Holst J Grote R Siegwolf R Kayler Z Gessler A The long way down—are carbon and oxygen isotope signals in the tree ring uncoupled from canopy physiological processes? Tree Physiol 2011 31 1088 1102 10.1093/treephys/tpr093 21957095

[B19] Kolb KJ Evans RD Implications of leaf nitrogen recycling on the nitrogen isotope composition of deciduous plant tissues New Phytol 2003 156 57 64

[B20] Evans RD Bloom AJ Sukrapanna SS Ehleringer JR Nitrogen isotope composition of tomato (*Lycopersicon esculentum* Mill. Cv. T-5) grown under ammonium or nitrate nutrition Plant Cell Environ 1996 19 1317 1323 10.1111/j.1365-3040.1996.tb00010.x

[B21] Marmann P Wendler R Millard P Heilmeier H Nitrogen storage and remobilization in ash (*Fraxinus excelsior*) under field and laboratory conditions Trees-Struct Funct 1997 11 298 305

[B22] Bollmark L Sennerby-Forsse L Ericsson T Seasonal dynamics and effects of nitrogen supply rate on nitrogen and carbohydrate reserves in cutting-derived *Salix viminalis* plants Can J Forest Res 1999 29 85 94 10.1139/x98-183

[B23] Coleman GD Physiology and regulation of seasonal nitrogen cycling in woody plants J Crop Improv 2004 10 237 259 10.1300/J411v10n01_10

[B24] Millard P Grelet GA Nitrogen storage and remobilization by trees: ecophysiological relevance in a changing world Tree Physiol 2010 30 1083 1095 10.1093/treephys/tpq042 20551251

[B25] Rossato L Laine P Ourry A Nitrogen storage and remobilization in *Brassica napus* L. during the growth cycle: nitrogen fluxes within the plant and changes in soluble protein patterns J Ex Bot 2001 52 1655 1663 10.1093/jexbot/52.361.1655 11479330

[B26] Soolanayakanahally RJ Guy RD Silim SN Drewes EC Schroeder WR Enhanced assimilation rate and water use efficiency with latitude through increased photosynthetic capacity and internal conductance in balsam poplar(*Populus balsamifera* L.) Plant Cell Environ 2009 32 1821 1832 10.1111/j.1365-3040.2009.02042.x 19712064

[B27] Johnson CM Stout PR Broyer TC Carlton AB Comparative chlorine requirements of different plant species Plant Soil 1957 8 337 353 10.1007/BF01666323

[B28] Solorzano L Determination of ammonia in natural waters by the phenol-hypochlorite method Limnol Oceanogr 1969 14 799 801 10.4319/lo.1969.14.5.0799

[B29] Cawse PA The determination of nitrate in soil solution by ultraviolet spectrometry Analyst 1967 92 311 315 10.1039/an9679200311

